# Effects of variable‐width jaw motion on beam characteristics for Radixact Synchrony®

**DOI:** 10.1002/acm2.13234

**Published:** 2021-03-29

**Authors:** William S. Ferris, Wesley S. Culberson, Jennifer B. Smilowitz, John E. Bayouth

**Affiliations:** ^1^ Department of Medical Physics School of Medicine and Public Health University of Wisconsin‐Madison Madison WI USA; ^2^ Department of Human Oncology School of Medicine and Public Health University of Wisconsin‐Madison Madison WI USA

**Keywords:** radixact, synchrony, tomotherapy

## Abstract

**Purpose:**

Radixact Synchrony corrects for target motion during treatment by adjusting the jaw and MLC positions in real time. As the jaws move off axis, Synchrony attempts to adjust for a loss in output due to the un‐flattened 6 MV beam by increasing the jaw aperture width. The purpose of this work was to assess the impact of the variable‐width aperture on delivered dose using measurements and simulations.

**Methods:**

Longitudinal beam profile measurements were acquired using an Edge diode with static gantry. Jaw‐offset peak, width, and integral factors were calculated for profiles with the jaws in the extreme positions using both variable‐width (Synchrony) and fixed‐width apertures. Treatment plans with target motion and compensation were compared to planned doses to study the impact of the variable aperture on volumetric dose.

**Results:**

The jaw offset peak factor (JOPF) for the Synchrony jaw settings were 0.964 and 0.983 for the 1.0‐ and 2.5‐cm jaw settings, respectively. These values decreased to 0.925 and 0.982 for the fixed‐width settings, indicating that the peak value of the profile would decrease by 7.5% compared to centered if the aperture width was held constant. The IMRT dose distributions reveal similar results, where gamma pass rates are above tolerance for the Synchrony jaw settings but fall significantly for the fixed‐width 1‐cm jaws.

**Conclusions:**

The variable‐width behavior of Synchrony jaws provides a larger output correction for the 1‐cm jaw setting. Without the variable‐aperture correction, plans with the 1‐cm jaw setting would underdose the target if the jaws spend a significant amount of time in the extreme positions. This work investigated the change in delivered dose with jaws in the extreme positions, therefore overall changes in dose due to offset jaws are expected to be less for composite treatment deliveries.

## 1 INTRODUCTION

The Radixact is a linear accelerator (linac) capable of delivering helical tomotherapy using an un‐flattened 6‐MV beam.^1^ The beam is collimated in the IEC‐Y direction (superior/inferior) using jaws and is collimated in the transverse direction using a 64‐leaf multi‐leaf collimator (MLC). The MLC leaves are binary, meaning they are set to be fully open or closed. For helical deliveries, the patient translates through the bore while the linac simultaneously rotates around the patient delivering radiation in slices. The MLC pattern at each gantry angle is called a sinogram, which is optimized to create an intensity‐modulated radiation therapy (IMRT) treatment.

**Fig. 1:**
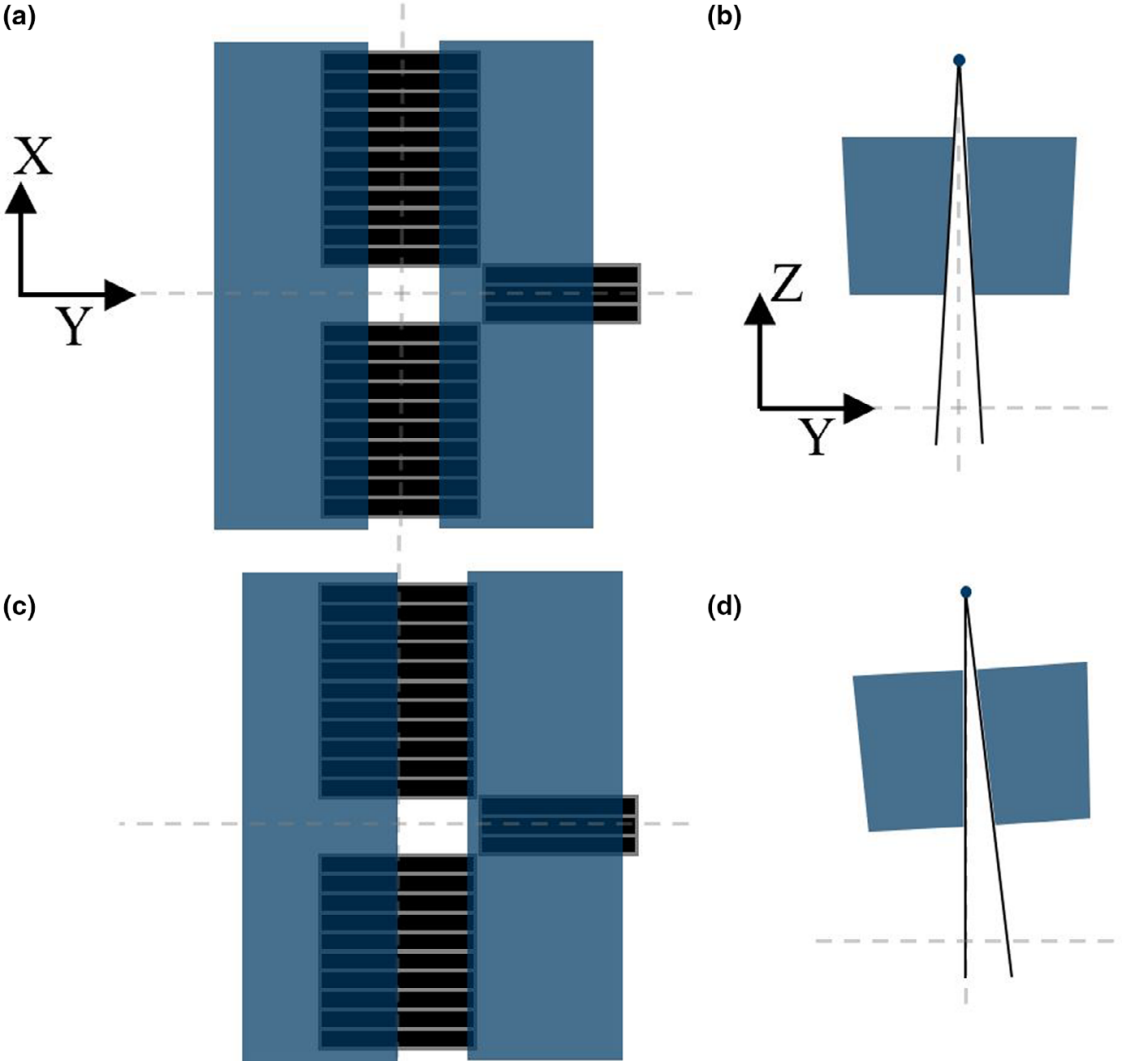
Schematic of collimation and jaw compensation on Radixact. Beam’s eye view (a) and side view (b) with centered jaws and central three leaves open. Beam’s eye view (c) and side view (d) with jaws compensating in the positive IEC‐Y direction. Note that there are 64 total MLC leaves on Radixact and drawings are not to scale.

Radixact Synchrony is an intrafraction motion management technique that attempts to correct for target motion by adjusting the collimation of the radiation in real‐time during treatment.^2^ Figure 1 shows a schematic of the collimation and method of jaw compensation on Radixact. The jaws compensate for motion in the IEC‐Y (superior/inferior) direction, and the multi‐leaf collimator (MLC) compensates for motion in the transverse plane (IEC‐X [left/right] and IEC‐Z [anterior/posterior] directions). A description of the Synchrony system has been provided by others in previous publications.^1^, ^2^, ^3^, ^4^, ^5^, ^6^

The Radixact uses an un‐flattened 6‐MV beam for helical tomotherapy treatment delivery. The un‐flattened beam profile is not an issue during standard intensity‐modulated radiation therapy (IMRT) treatments because the change in fluence off‐axis is accounted for in the treatment planning system. However, when the collimation follows the target during a Synchrony treatment, the variation in distance from the beam central axis introduces an un‐planned change in output. The method currently implemented by Accuray to correct for this reduced output is to increase the jaw width, with corrections of output only being implemented in the IEC‐Y direction. Motion in the IEC‐X and IEC‐Z directions for lung and upper abdominal targets has been observed to be much smaller than motion in the IEC‐Y direction.^7^, ^8^, ^9^ Since the jaws are always centered in the original treatment plan (excluding dynamic jaw patterns), the fluence always decreases for jaw motion shifts in the positive or negative IEC‐Y direction. However, changes in fluence due to motion in the axial plane are more complex since shifts in IEC‐X and IEC‐Z may move the target closer or farther from the central axis.

Theoretical methods of correcting for a loss of output include increasing the dose rate, decreasing the rotation speed, and increasing the leaf open time for a given projection (if the leaf is not already open for the full projection). However, these methods are difficult to implement during treatment delivery due to mechanical restrictions and the need to account for the latency in target position prediction.

Chen et al investigated the effects of offset jaws on beam characteristics using metrics such as jaw‐offset peak factor (JOPF) and jaw‐offset width factor (JOWF).^3^ These values were derived from longitudinal beam profiles measured using an A1SL ion chamber (Standard Imaging Inc, Middleton, WI) aligned perpendicular to the direction of table travel and inside a rectangular virtual water stack. However, the measurements were performed using a fixed aperture width as the jaws move off‐axis, which is appropriate for machine quality assurance measurements, but is not representative of how the jaws behave during a Synchrony treatment where the aperture width changes. In addition, the A1SL has a 4‐mm active volume diameter, which may cause changes to the peak and width of the profiles, especially for the small field width of 10 mm.

The purpose of this work was to investigate the changes in beam characteristics and dose accuracy during a Synchrony treatment due to variable‐width jaw movement. The worst‐case scenarios have been investigated with the jaws offset to the extreme positions. Beam characteristics during a Synchrony treatment were compared to those that would result if the beam width were kept constant rather than variable as the jaws move off‐axis. Analysis was performed using profile measurements and simulated helical treatment plans.

## 2 METHODS

### 2.1 Longitudinal profiles

**Fig. 2:**
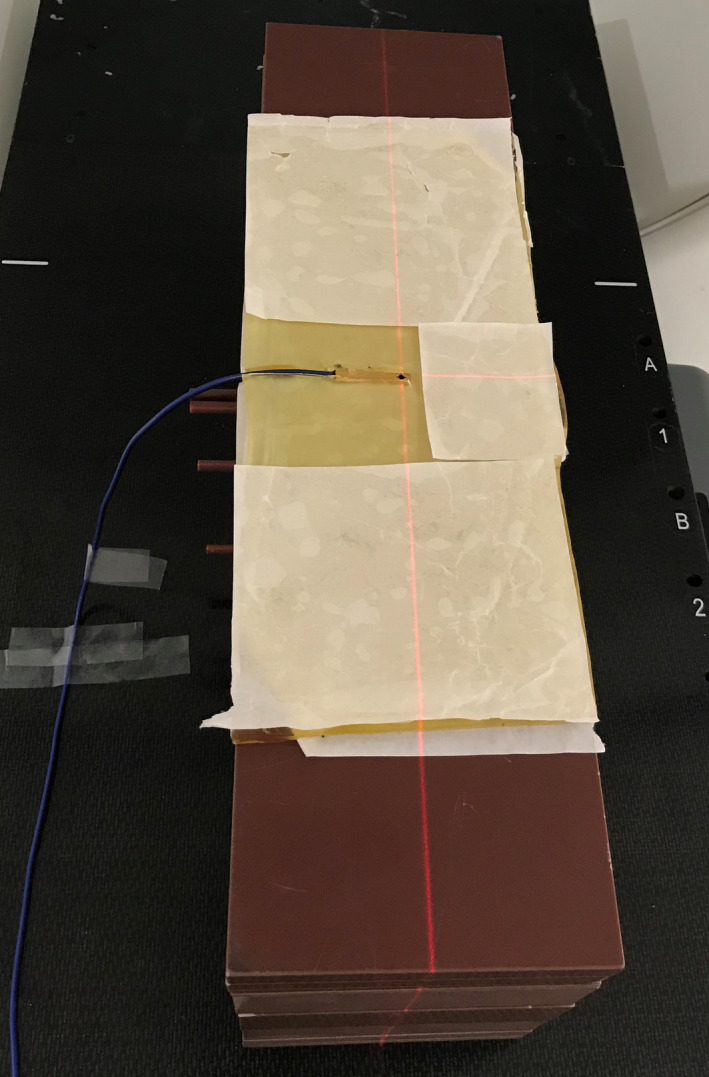
Photograph of the measurement setup with Edge diode aligned perpendicular to the direction of couch travel. A cutout was made in the 5 mm thick bolus material to reduce the air gap around the detector. The 10 cm of solid water on top of the gel and detector was removed for the photograph. The detector volume was centered in IEC‐X.

Longitudinal beam profiles were measured for the 2.5‐cm and 1‐cm jaw settings using an Edge diode detector (active area 0.8 × 0.8 mm; Sun Nuclear, Melbourne, FL) for the jaws at the centered, positive extreme, and negative extreme positions possible for Synchrony motion tracking. The detector long axis was aligned perpendicular to the direction of table travel inside a rectangular virtual water stack and the profiles were acquired topographically (static beam and couch translating through bore), shown in Fig. 2. A cutout was made in a 5‐mm‐thick flexible bolus material of unit density to hold the detector and reduce the air gap. Rectangular virtual water of 10‐cm thickness was placed on top of the bolus and detector such that the source to phantom surface was 85 cm and the surface of the detector was positioned at a depth of 10 cm. Profiles were only measured at one depth (10 cm) as Chen et al. reported no significant change in beam characteristics with depth.^3^ The gantry was set to 0° (beam pointing toward the floor) with all MLC leaves open and the table translated through the bore at 0.5 mm/s. Profiles were smoothed with a 3 mm moving average filter to remove variations in the peak value.

The jaw settings available for Synchrony on the Radixact are 2.5 and 1.0 cm, which refer to the approximate full‐width half‐maximum (FWHM) of the dose profile at 1.5‐cm depth in water at isocenter. These jaw settings correspond to physical aperture widths of 2.0 cm and 0.7 cm, respectively, as the distal surface of the collimator is approximately 23 cm from the target. (Tomotherapy can also be delivered with a 5.0‐cm jaw width but this jaw setting is not available for Synchrony treatments since the jaws cannot open larger than the nominal 5‐cm widest field.) The majority of non‐Synchrony helical tomotherapy plans in our clinic use the 2.5‐cm jaw setting. However, the 1‐cm jaw setting may be used more commonly for Synchrony treatments since the range of motion compensation is larger (4 cm compared to 2.5 cm).

For the off‐axis positions, profiles were measured with two aperture settings with distinct widths, shown in Table 1. For the 2.5‐cm jaw setting, the jaws were shifted as if they were compensating for a shift in the target location of ±1.25 cm at isocenter, which is the maximum magnitude of motion in IEC‐Y that can be compensated by Synchrony. Similarly, for the 1.0 jaw setting, the jaws were shifted as if compensating for a shift at isocenter of ±2 cm in IEC‐Y. For Synchrony, the jaw shifts include an increase in aperture width, as shown in Table 1. To investigate the effect of increasing the aperture width on the beam characteristics, profiles were also measured with the front and back jaws shifted the same amount such that aperture width stays constant. This is denoted “fixed‐width”. Note – in conventional (non‐Synchrony) tomotherapy delivery, there are two jaw‐setting options, fixed‐width and dynamic (aka running start and stop, or RSS). This refers to whether or not the jaws open and close gradually at the start and end of the field length to sharpen the superior and inferior field edges. In this work, “fixed‐width” refers to a simplification of how the jaws compensate for motion during Synchrony, and not to the behavior of the jaws at the start and end of treatment.

**Table 1:** Jaw aperture widths used in this work. Aperture settings refer to the physical aperture at 23 cm from the MV target. The extreme locations correspond to shifts in IEC‐Y at isocenter of ± 2.0 cm and ± 1.25 cm for the 7‐mm and 20‐mm apertures, respectively.

	Jaw aperture width (mm)		
Compensation mode	Centered	Positive extreme	Negative extreme
Fixed‐width	7.00	7.00	7.00
Synchrony	7.00	7.75	7.75
Fixed‐width	20.00	20.00	20.00
Synchrony	20.00	20.15	20.15

The profiles were also calculated using the Precision treatment planning system (TPS; Accuray Inc, Sunnyvale, CA) using the same setup conditions (85‐cm SSD, 10‐cm depth, etc.). The resolution of the dose calculation was 0.2 mm in the IEC‐Y direction and 1 mm in the IEC‐X and IEC‐Z directions (the resolution in IEC‐Y was made as fine as the dose calculation engine could handle).

Three characteristics were calculated for each profile: JOPF, JOWF and jaw‐offset integral factor (JOIF). The peak value of each profile is denoted *D_max_* and the FWHM of each profile in millimeters is denoted *W*. Widths were calculated using linear interpolation. The jaw setting of the profile is denoted *J*, which was either 2.5 cm or 1 cm. The compensation mode for the offset‐jaw profiles is denoted *S* or *F*, for “Synchrony”, or “Fixed‐width”. The location of the center of the aperture in IEC‐Y projected to isocenter is denoted *Y_jaw_*, which is 0 cm for the centered jaws and ± 2 cm or ± 1.25 cm for the 1‐cm and 2.5‐cm jaw settings, respectively. The integral of the profile is denoted *I*, which is a function of the threshold, *T*, used for integration (e.g., 10% threshold). The depth is 10 cm for all profiles.

(1) $$\text{JOPF}=\frac{D_{\max}\left(J,Y_{\mathrm{jaw}},S/F\right)}{D_{\max}\left(J,0\right)}$$

(2) $$\text{JOWF}=\frac{W\left(J,Y_{\mathrm{jaw}},S/F\right)}{W\left(J,0\right)}$$

(3) $$\text{JOIF}=\frac{I\left(J,Y_{\mathrm{jaw}},T,S/F\right)}{I\left(J,0,T\right)}$$

### 2.2 IMRT treatment plan simulation

Longitudinal profiles provide insight into the change in the shape of the dose profile for a given projection, but they do not describe the change in the volumetric dose distribution over many helical rotations. Therefore, the effect of the change in jaw aperture width on the volumetric dose distribution was explored using simulated treatment plans. A cylindrical target (5‐cm diameter, 5‐cm length) was created at the center of a cylindrical phantom (30‐cm diameter, 30‐cm length) of water aligned along the IEC‐Y direction. The phantom was centered in IEC‐X and IEC‐Z. Treatment plans were generated using Precision with both the 2.5‐cm and 1‐cm jaw settings. The IMRT plans were optimized to deliver 50 Gy to 98% of the volume in five fractions with a steep dose gradient at the edge of the target. This hypo‐fractionated treatment schedule was chosen since hypofractionated stereotactic body radiotherapy (SBRT) deliveries require more precise localization and this fractionation scheme has been used for Synchrony treatments for lung cancer published in the literature.^4^, ^7^ Dose calculation used a convolution/superposition algorithm.

The effect of motion compensation was explored by comparing the planned dose to dose resulting from a target shift in IEC‐Y with corresponding jaw shifts to provide compensation. The target was simulated to shift by the same amount as used for the longitudinal profiles in Section 2.1 (±1.25 cm and ±2 cm in IEC‐Y for the 2.5‐cm and 1‐cm jaw plans, respectively) and the same jaw aperture widths settings as the longitudinal profiles were used, shown in Table 1. The target was assumed to shift by the specified amount and stay there throughout treatment, creating a “worst‐case” impact of the un‐flattened beam profile in which the jaws are always shifted to their negative or positive extreme locations throughout treatment. The effect of the variable‐width aperture used by Synchrony was investigated by comparing the plan with the simulated Synchrony jaw behavior to a plan with a fixed‐width jaw.

The plans were analyzed using IEC‐Y profiles and gamma analysis. The voxel size of the dose calculation for the helical plans was 0.5 × 1 × 1 mm^3^.

## 3 RESULTS

**Fig. 3:**
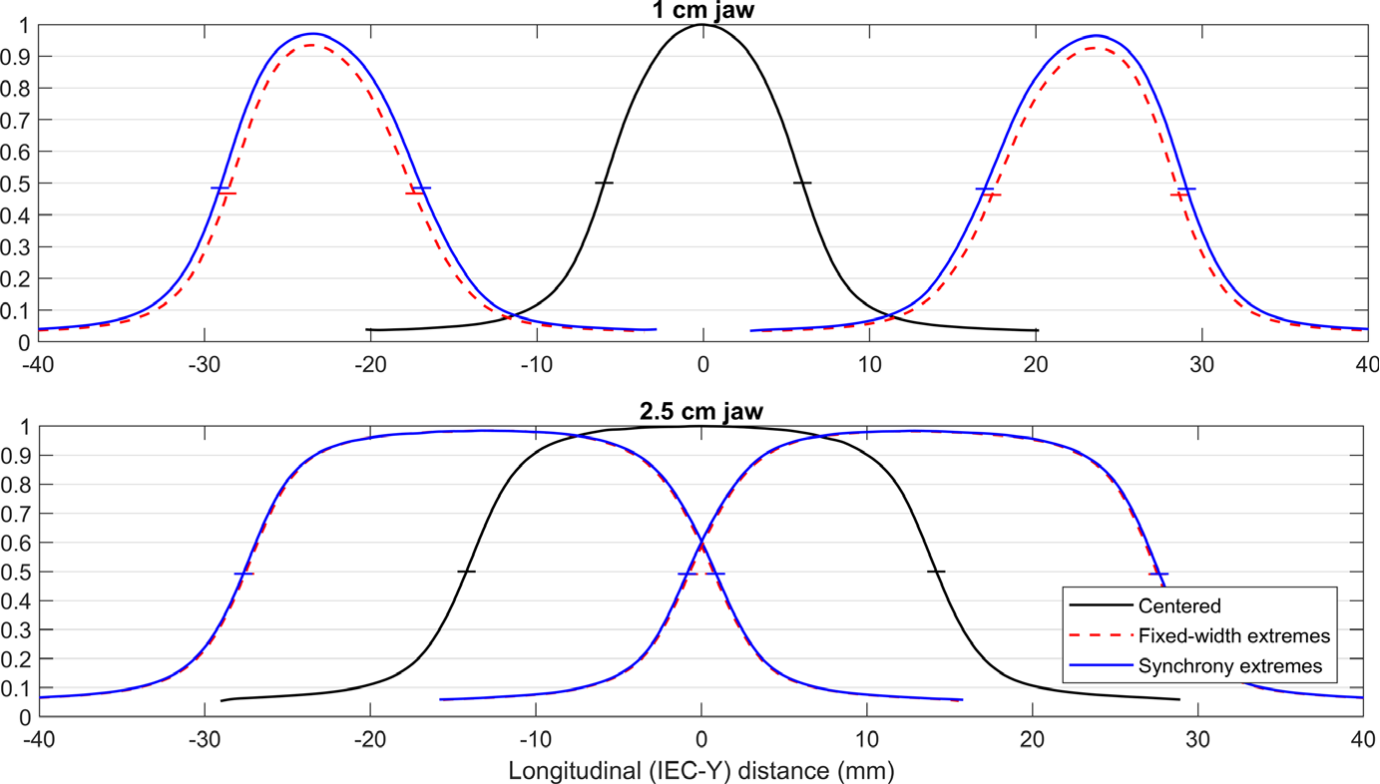
Profiles at 10‐cm depth and 85‐cm source‐to‐surface distance measured with an Edge diode. The y‐axis displays the signal relative to the maximum signal for the profile measured with the centered aperture. The colored horizontal ticks indicate the level of the FWHM for each curve. Displayed at the off‐axis locations are profiles acquired with varying jaw aperture widths (see Table 1).

Figure 3 shows the longitudinal profiles measured with the Edge diode for the Synchrony and fixed‐width settings. The JOPF, JOWF, and JOIF are derived from the profiles and are listed in Table 2. The width of the centered‐jaw profiles at 10‐cm depth were 2.84 cm and 1.19 cm for the 2.5‐cm and 1.0‐cm jaw settings, respectively, and the JOWFs are relative to these values. The reduction in the peak value relative to the centered profile can be visually observed in the profiles, especially those for the 1‐cm jaw and the fixed‐width setting.

**Table 2:** Beam characteristics (jaw‐offset peak, width, and integral factors) for measured profiles at 10‐cm depth. The JOIFs were calculated with a 10% dose threshold. The pair of values in each entry indicates the value for the negative extreme and positive extreme profile, respectively.

Jaw	Compensation type	JOPF	JOWF	JOIF
2.5 cm	Synchrony	0.984/0.983	1.007/1.006	0.978/0.976
	Fixed‐width	0.984/0.982	0.999/0.998	0.981/0.978
1.0 cm	Synchrony	0.970/0.964	1.019/1.020	0.992/0.983
	Fixed‐width	0.934/0.925	0.944/0.945	0.894/0.883

The longitudinal profiles were also calculated using the Precision TPS to verify the accuracy of the treatment planning system for changes in beam output and width for offset jaws. The median absolute difference between the simulated and measured JOPF was 0.2% (max 1.6%). The median difference between the simulated and measured JOWF was 0.2% (max 0.5%). The median difference between the simulated and measured JOIF was 0.5% (max 1.3%).

**Fig. 4:**
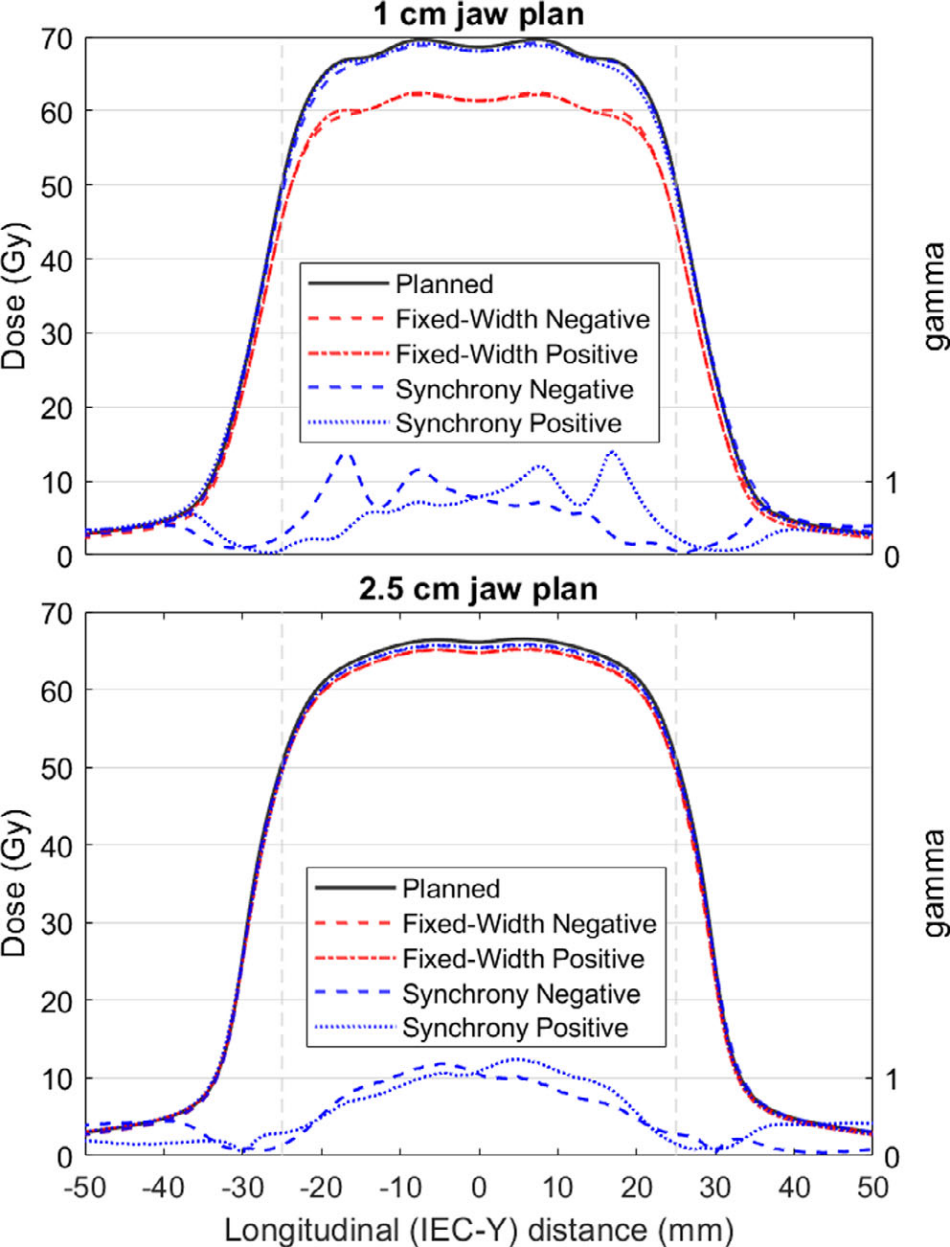
Simulated dose profiles in the IEC‐Y direction in the frame of reference of a cylindrical target from a helical IMRT delivery. Profiles are centered in X and Z. The vertical lines indicate the limits of the target in IEC‐Y (5‐cm diameter, 5‐cm length cylinder). The prescription dose was 50 Gy. Negative and positive refer to the target shifted to the positive or negative extreme location throughout treatment and compensated with jaw motion. The resolution is 0.5 mm in the IEC‐Y direction. Gamma profiles (1%, 1 mm) are shown for the Synchrony plans compared to planned.

Simulated longitudinal profiles in IEC‐Y for both the 2.5‐cm and 1‐cm jaw treatment plans through the center of the cylinder are shown in Fig. 4. Volumetric gamma analyses comparing the Synchrony‐compensated and planned dose distributions are shown in Table 3. Two separate gamma analysis criteria were used: 3%, 2 mm, 10% threshold and 1%, 1 mm, 10% threshold.

**Table 3:** Gamma pass rates for simulated treatment plans comparing the motion compensated dose distributions to planned. All dose differences are relative to the global maximum dose. A dose threshold of 10% of the prescription dose (50 Gy) is applied to all data. Gamma analyses are 3D. The extreme locations correspond to shifts in IEC‐Y at isocenter of ± 2.0 cm and ± 1.25 cm for the 1.0‐cm and 2.5‐cm jaws, respectively.

Jaw	Position	Fixed‐Width		Synchrony	
		3%, 2 mm^a^	1%, 1 mm	3%, 2mm^a^	1%, 1 mm
2.5 cm	Negative extreme	100	94.67	100	99.20
	Positive extreme	100	94.44	100	98.70
1.0 cm	Negative extreme	62.16	48.11	100	98.89
	Positive extreme	62.01	47.02	100	99.05

^a^ Universal tolerance limit recommended by TG‐218 for IMRT QA is > 95% of points passing 3%, 2 mm, with a 10% dose threshold.^10^

## 4 DISCUSSION

The profile experiments in this work provided a worst‐case scenario in terms of the effect of the un‐flattened beam since characteristics were measured or simulated with the jaws in the extreme off‐axis positions. For the 1‐cm jaw setting, the maximum reduction in peak value due to offset jaws that can be expected with Synchrony jaw behavior is 3.6%. However, this reduction in peak value will be accompanied by an increase in width of 2%. The integral of the profile stays within 1.7% of the integral of the centered profile. For the 2.5‐cm jaw, the maximum reduction in peak value that can be expected with Synchrony jaw behavior is 1.7%, which is accompanied by an increase in field width of 0.6%. One must note that these results are for maximally offset jaws. The target may move farther from centered in the IEC‐Y direction than this, which may cause the treatment to be paused.

The profiles measured with the fixed‐width approximation provide insight on the effect of the increase of the aperture width that is characteristic of jaws during a Synchrony treatment. For the 1‐cm jaw and fixed‐width jaw behavior, the peak value was observed to decrease by 7.5% (compared to 3.6% for Synchrony). For the 2.5‐cm jaw setting, there is less of an effect of the increase in jaw width used by Synchrony. The peak value for the fixed‐width profiles stays within 1.8% of the centered profile peak (compared to 1.7% for Synchrony). The values measured with the fixed‐width approximation agree with the values reported by Chen et al.^3^ and would represent the decrease in output if the vendor had not applied the Synchrony jaw output correction.

For the IMRT plans with Synchrony jaw compensation, the gamma pass rates between the compensated dose and the planned dose are all 100% when using criteria recommended by TG‐218 for IMRT (3%, 2 mm, 10% threshold).^10^ For more strict criteria (1%, 1 mm, 10% threshold), the pass rates decrease slightly but are still 98.5% or greater. In the longitudinal profiles through the target in Fig. 4, a minor underdosing (~1–2%) can be observed in the Synchrony‐compensated doses (blue lines) relative to the planned dose.

The underdosing of the fixed‐aperture doses compared to the planned dose is much more apparent, especially with the 1‐cm jaws. The 2.5‐cm jaw plan with the fixed‐width aperture is still above the tolerance limit of 95% for recommended IMRT criteria (3%, 2 mm, 10% threshold). However, the gamma pass rates are less than 95% for more strict criteria (1%, 1 mm), while they were >98% for the variable‐width aperture of Synchrony. This confirms the finding from the longitudinal profile measurements that for the 2.5‐cm jaw criteria, the variable‐width nature of the jaws during Synchrony is not entirely necessary, but does improve agreement with the planned dose slightly. Conversely, the gamma pass rates for the 1‐cm plan and the fixed‐width approximation are well below 95%, and the gross underdosing is apparent in Fig. 4. For standard criteria (3%, 2 mm), the gamma pass rates drop from 100% with the variable‐width aperture used by Synchrony to ~62% with the fixed‐width aperture.

**Fig. 5:**
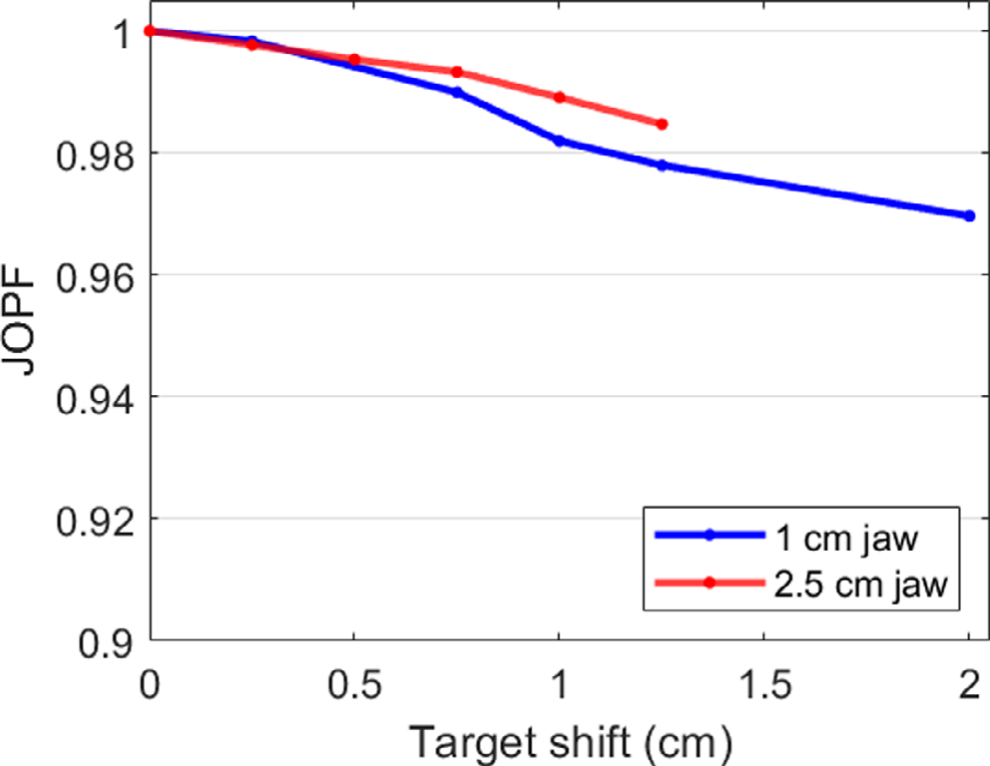
Jaw‐offset peak factors (JOPFs) as a function of target shift in IEC‐Y for the variable‐aperture jaw behavior during Synchrony treatments. Profiles were calculated with the TPS at a depth of 1.5‐cm and 85‐cm SSD. The values are an average of positive and negative JOPFs. Values at the extreme positions agree with the measured values in Table 2.

Figure 5 displays the change in JOPF as a function of off‐axis position. Data presented previously in this work were acquired for extreme jaw positions in order to provide clinical users an estimate of the maximum induced changes from the un‐flattened beam and off‐axis jaw motion. However, Figure 5 can be used to estimate the change in peak profile value for target shifts that are less than the maximum shifts. For complex target motions, reduction in output reaching the target due to the un‐flattened beam throughout treatment can be estimated by calculating the average absolute target offset and looking up JOPF in Figure 5.

## 5 CONCLUSIONS

Longitudinal profile measurements and simulated treatment plans were used to investigate the effect of the variable‐width jaws that is characteristic of Synchrony. Longitudinal profiles indicate that the maximum decrease in output due to jaw sway for Synchrony is on the order of 3.5% and 2% for the 1‐cm and 2.5‐cm jaw settings, respectively. These values would be 7.5% and 2% if the jaws moved off axis but kept a fixed width. The simulated IMRT treatments with Synchrony jaw compensation indicate that gamma pass rates are clinically acceptable even with maximally offset jaws. Pass rates decreased significantly for the fixed‐width aperture for the 1‐cm jaws.

Extreme jaw positions were considered for this work to provide clinical users with the maximum effect that may be observed due to off axis jaw positions. For realistic patient motion, the target is unlikely to be in the maximally offset IEC‐Y position throughout treatment, therefore the changes in dose due to off‐axis fields are expected to be smaller than indicated in this work. This work presented a method (Figure 5) to estimate JOPF for motions less than the extremes.

## Conflict of interest

The authors have no conflicts of interest to disclose.
