# Erratum: “Effects of variable‐width jaw motion on beam characteristics for Radixact Synchrony®”

**DOI:** 10.1002/acm2.13883

**Published:** 2023-02-16

This erratum corrects the following:

Ferris WS, Culberson WS, Smilowitz JB, Bayouth JE. Effects of variable‐width jaw motion on beam characteristics for Radixact Synchrony®. *J Appl Clin Med Phys*. 2022; 22: 175–181. https://doi.org/10.1002/acm2.13234

## CONFLICT OF INTEREST

John E. Bayouth reports ownership interest in a company that provides consulting services on image‐guided radiation therapy technology (MR Guidance, LLC,). He/(his employer) received travel reimbursement/speaking fees from ViewRay Inc. The remaining authors have no conflicts of interest to disclose.

